# Supplementary material for: Causal Relationship between Aging and Anorexia Nervosa: A White-Matter-Microstructure-Mediated Mendelian Randomization Analysis
Source: Biomedicines. 2024 Aug 16;12(8):1874. doi: 10.3390/biomedicines12081874 (PMC11351342; doi:10.3390/biomedicines12081874)
Supplement: Supplementary file 1 [file biomedicines-12-01874-s001.zip › Supplementary Figure/Figure S4/Singe SNP plot of MR analysis for TL to ubm-b-1966.pdf]

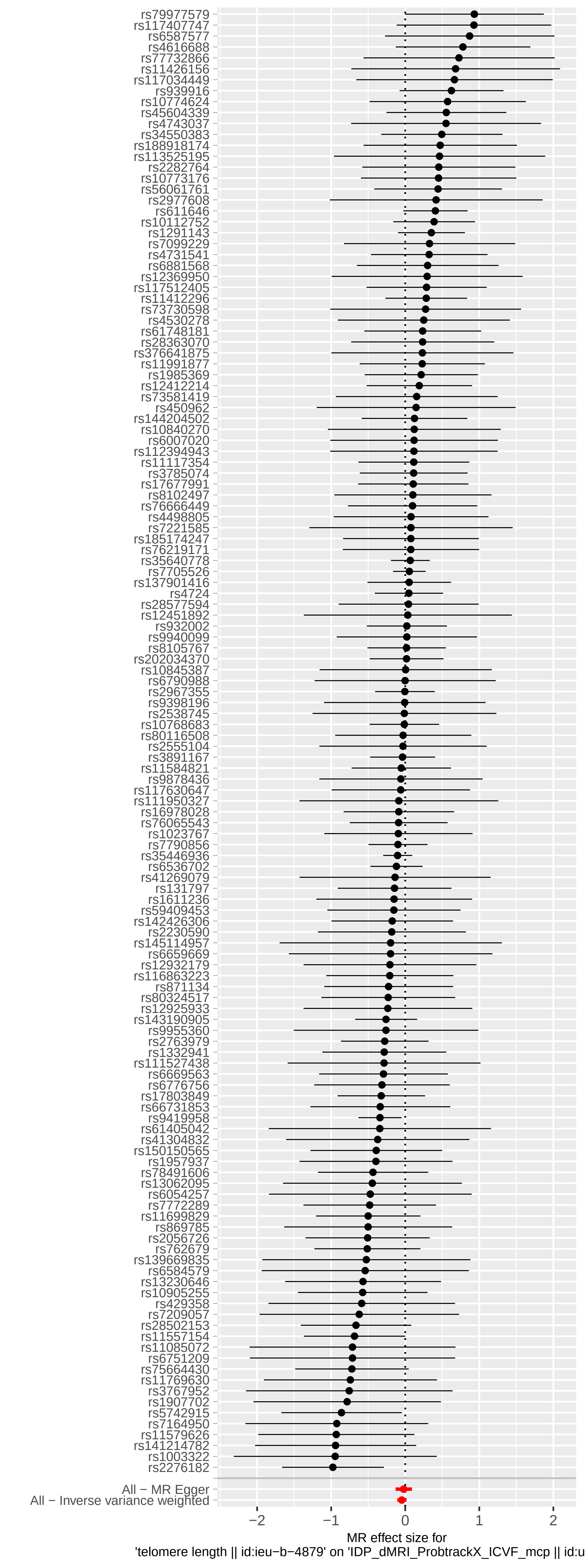

rs79977579
rs117407747
rs6587577
rs4616688
rs77732866
rs11426156
rs117034449
rs939916
rs10774624
rs45604339
rs4743037
rs34550383
rs188918174
rs113525195
rs2282764
rs10773176
rs56061761
rs2977608
rs611646
rs10112752
rs1291143
rs7099229
rs4731541
rs6881568
rs12369950
rs117512405
rs11412296
rs73730598
rs4530278
rs61748181
rs28363070
rs376641875
rs11991877
rs1985369
rs12412214
rs73581419
rs450962
rs144204502
rs10840270
rs6007020
rs112394943
rs11117354
rs3785074
rs17677991
rs8102497
rs76666449
rs4498805
rs7221585
rs185174247
rs76219171
rs35640778
rs7705526
rs137901416
rs4724
rs28577594
rs12451892
rs932002
rs9940099
rs8105767
rs202034370
rs10845387
rs6790988
rs2967355
rs9398196
rs2538745
rs10768683
rs80116508
rs2555104
rs3891167
rs11584821
rs9878436
rs117630647
rs111950327
rs16978028
rs76065543
rs1023767
rs7790856
rs35446936
rs6536702
rs41269079
rs131797
rs1611236
rs59409453
rs142426306
rs2230590
rs145114957
rs6659669
rs12932179
rs116863223
rs871134
rs80324517
rs12925933
rs143190905
rs9955360
rs2763979
rs1332941
rs111527438
rs6669563
rs6776756
rs17803849
rs66731853
rs9419958
rs61405042
rs41304832
rs150150565
rs1957937
rs78491606
rs13062095
rs6054257
rs7772289
rs11699829
rs869785
rs2056726
rs762679
rs139669835
rs6584579
rs13230646
rs10905255
rs429358
rs7209057
rs28502153
rs11557154
rs11085072
rs6751209
rs75664430
rs11769630
rs3767952
rs1907702
rs5742915
rs7164950
rs11579626
rs141214782
rs1003322
rs2276182
All − MR Egger
All − Inverse variance weighted
−2
−1
0
1
2
MR effect size for
'telomere length || id:ieu-b-4879' on 'IDP_dMRI_ProbtrackX_ICVF_mcp || id:u
